# Oil type and temperature dependent biodegradation dynamics - Combining chemical and microbial community data through multivariate analysis

**DOI:** 10.1186/s12866-018-1221-9

**Published:** 2018-08-07

**Authors:** Deni Ribicic, Kelly Marie McFarlin, Roman Netzer, Odd Gunnar Brakstad, Anika Winkler, Mimmi Throne-Holst, Trond Røvik Størseth

**Affiliations:** 1 SINTEF Ocean, Environment and New Resources, Brattørkaia 17C, 7010 Trondheim, Norway; 2 Department Clinical and Molecular Medicine, The Norwegian University of Science and Technology, 7491 Trondheim, Norway; 3 Bielefeld University, Center for Biotechnology (CeBiTec), 33501 Bielefeld, Germany

## Abstract

**Background:**

This study investigates a comparative multivariate approach for studying the biodegradation of chemically dispersed oil. The rationale for this approach lies in the inherent complexity of the data and challenges associated with comparing multiple experiments with inconsistent sampling points, with respect to inferring correlations and visualizing multiple datasets with numerous variables. We aim to identify novel correlations among microbial community composition, the chemical change of individual petroleum hydrocarbons, oil type and temperature by creating modelled datasets from inconsistent sampling time points. Four different incubation experiments were conducted with freshly collected Norwegian seawater and either Grane and Troll oil dispersed with Corexit 9500. Incubations were conducted at two different temperatures (5 °C and 13 °C) over a period of 64 days.

**Results:**

PCA analysis of modelled chemical datasets and calculated half-lives revealed differences in the biodegradation of individual hydrocarbons among temperatures and oil types. At 5 °C, most *n*-alkanes biodegraded faster in heavy Grane oil compared to light Troll oil. PCA analysis of modelled microbial community datasets reveal differences between temperature and oil type, especially at low temperature. For both oils, *Colwelliaceae and Oceanospirillaceae* were more prominent in the colder incubation (5 °C) than the warmer (13 °C). Overall, *Colwelliaceae*, *Oceanospirillaceae*, *Flavobacteriaceae*, *Rhodobacteraceae*, *Alteromonadaceae* and *Piscirickettsiaceae* consistently dominated the microbial community at both temperatures and in both oil types. Other families known to include oil-degrading bacteria were also identified, such as *Alcanivoracaceae, Methylophilaceae, Sphingomonadaceae* and *Erythrobacteraceae*, but they were all present in dispersed oil incubations at a low abundance (< 1%).

**Conclusions:**

In the current study, our goal was to introduce a comparative multivariate approach for studying the biodegradation of dispersed oil, including curve-fitted models of datasets for a greater data resolution and comparability. By applying these approaches, we have shown how different temperatures and oil types influence the biodegradation of oil in incubations with inconsistent sampling points. Clustering analysis revealed further how temperature and oil type influence single compound depletion and microbial community composition. Finally, correlation analysis of degraders community, with single compound data, revealed complexity beneath usual abundance cut-offs used for microbial community data in biodegradation studies.

**Electronic supplementary material:**

The online version of this article (10.1186/s12866-018-1221-9) contains supplementary material, which is available to authorized users.

## Background

Marine oil biodegradation is influenced by a variety of factors, both environmental (e.g. microbial composition, nutrient and oxygen concentrations, seawater temperature, and the presence of ice) and oil-related (e.g. oil type, concentration, and weathering). The majority of oil-biodegradation studies have focused on environmental effects, while the influence of oil type on biodegradation has not received as much attention. Environmental factors and oil properties do not affect oil-biodegradation separately, but have combined effects as environmental factors, such as temperature and wind, can have profound effects on the property of oil over time. To truly understand how environmental factors influence oil-biodegradation, it is important to study and compare many variables at once. Therefore, it becomes important to combine environmental factors with the physicochemical properties of oil when studying their effect on oil-biodegradation.

Temperature, and thus thermodynamics, influences all aspects of life [1] and has been shown to play a predominant role in oil biodegradation [2, 3]. Temperature influences the activity of microbial enzymes and the physicochemical characteristics of the oil. In general, biochemical reactions exponentially decrease with decreasing temperature but microorganisms have adapted mechanisms to thrive at low temperatures [4]. Psychrophilic microorganisms overcome constraints imposed by low temperatures by producing cold-adapted enzymes, which allow them to function more efficiently at low temperatures compared to their mesophilic counterparts [4, 5]. The ability of temperature to influence microbial community structure is well known [6], but field studies strongly suggest that oil-degrading potential is not influenced by statistical differences in microbial community structure. For example, the genetic potential to biodegrade oil was found to be independent of community structure in offshore Arctic seawater [7]. In addition, similar oil-degrading genes have been found in cold and temperate environments [7, 8]. The ubiquity of oil-degrading microorganisms highlights the abundance of genetic transfer and the importance of molecular analyses.

The effects of temperature on the physicochemical properties of oil is likely to have a stronger influence on oil-biodegradation than would the influence of microbial community structure. Declining temperatures increase oil viscosity, while decreasing rates of solubility, dissolution, and volatilization. These processes decrease the bioavailability of oil compounds to degradative microorganisms and may prolong the presence of toxic aromatic hydrocarbons in cold environments [2, 9].

Characterizing the biodegradation of oil through detailed chemical and microbial analysis is challenging due to the extremely large data sets produced with high throughput technology. Such large data sets are even responsible for the creation of a new field of study (i.e. bioinformatics), which develops computational tools to understand biological data [10]. The inherent complexity of these data represents challenges, both with respect to inferring correlations and the visualization of multiple datasets with hundreds to thousands of components (i.e. oil compounds or bacterial genes) and numerous environmental variables. The complexity of oil analysis is often alleviated by grouping compounds into classes [11]; however, this approach should be used with caution as grouping may mask trends in single component degradation. For microbial community data, a typical approach is to focus on the most abundant species, as they are expected to have the greatest effect on biodegradation.

Here, we demonstrate how simple statistical tools can be utilized to analyze complex data sets. Common statistical analyses, including ordinations and grouping classifications, can infer differences and allow the visualization of patterns. For single variable datasets, principal components analysis (PCA) is one of the most commonly used ordination tools and allows users to reduce the variation between data points while conserving dominant trends [12]. Data generated in such a way allows for direct comparison of one sample with another but limits the user to one variable. Here we use PCA plots and heatmaps to identify relationships describing the biodegradation of Troll and Grane oil at two different temperatures (5 °C and 13 °C). Another objective of this study is to predict the chemical loss of oil and the microbial community structure at different time points. This predictive analysis may save time and cost by quantitatively predicting oil loss at different temperatures and biodegradation potential in the absence of sampling. Here we compare two incubation studies with different sampling times to demonstrate how a predictive curve-fitted model enables comparisons between different treatments at the same time point in the absence of uniform sample collection.

## Methods

### Experiment setup

**Fig. 1:**
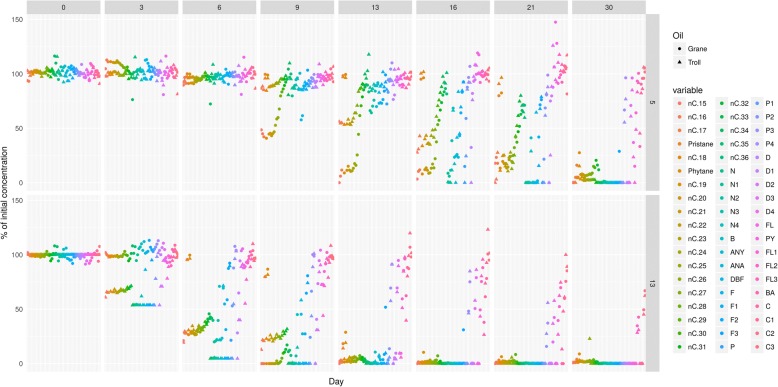
Complete chemistry dataset. Freshly collected seawater was incubated with two separate chemically dispersed oils (2–3 mg/L) at two different temperatures (13 °C and 5 °C). The change in concentration is shown on the y-axis as a percent of the initial concentration. The y-axis to the right identifies the treatment that each row illustrates. The treatments containing Grane oil are shown in the top two rows, with Grane 13 representing the mesocosms incubated at 13 °C and Grane 5 representing the mesocosms incubated at 5 °C. The treatments containing Troll oil are shown in the bottom two rows and follow the same labeling scheme. The experiment was conducted for 64 days and each column represents a different time point. Target compounds are shown in the legend and are represented as assorted colors. All time points were not analyzed for each treatment, which created gaps in the dataset and an opportunity to utilize predictive models

Two fresh crude oils (Grane and Troll), with different chemical properties, were chemically dispersed into freshly collected seawater. Mesocosms were incubated for 30–64 days in a carousel system at two temperatures (5 °C and 13 °C). Therefore, four different incubation experiments (two oils at two temperatures) were conducted and were sampled at different time points (Fig. 1 and Additional file 1: Table S1). At each time point (day 3, 6, 7, 9, 13, 14, 16, 21, 30 and 64) samples were collected and analyzed for chemical loss and microbial community structure.

### Oil, dispersant and seawater

Troll (naphthenic oil, 2007–0087) and Grane (asphaltenic oil, 2012–0331) were pre-heated at 50 °C for 30 min to melt the wax in the oil caused by cold storage, and then cooled to room temperature. Physicochemical characteristics of the two oils are shown in Table 1. The oils were premixed with Corexit 9500 (DOR 1:100) before preparing the oil/seawater dispersions (see next section). Seawater was collected via a pipeline system at 80 m depth (below thermocline, salinity 34 ‰, 5.9 °C) in a Norwegian fjord (Trondheimsfjord; 63°26’N, 10°23′E), outside the harbour area of Trondheim and was acclimated for two days at 5 °C and 13 °C before start of the biodegradation experiment.

**Table 1:** Properties of Troll and Grane oil

Oil	Category	Viscosity (mPas 13 °C)	Density (g/cm3)	Pour point (°C)	Wax (vol%)	Asphaltene (wt%)
Troll	Naphthenic	27	0.900	−18	2.0	0.20
Grane	Asphaltenic	667	0.941	−18	1.5	1.40

### Biodegradation experiment

Stock dispersion solutions were created with a droplet generator system [13] which ensured 10 μm oil droplets and an oil concentration of 200 mg/L. Based on oil droplet concentration measurements (Coulter Counter; see next section), each stock dispersion was diluted in natural unfiltered seawater (previously acclimated at 5 °C and 13 °C) to reach a final nominal concentration of 2–3 mg/L oil droplets. Negative controls contained sterilized (HgCl_2_) seawater. Additional nutrients were not added to the natural seawater as previous studies have shown that these low oil concentrations do not produce limiting conditions [14, 15]. The seawater dispersions were distributed in baked (450 °C) and autoclaved flasks (2 L; Schott), and were filled and capped without headspace or air bubbles. Flasks were mounted on a carousel system with slow continuous rotation (0.75 rpm), as previously described [13]. The carousels were maintained at 5 °C and 13 °C for 64 days in the dark, and flasks were sacrificed in replicates for analyses after 30 min incubation. Sampling regimes and replicates are described in detail in Additional file 1: Table S1. In addition to the sterilized controls, seawater controls containing unfiltered seawater without oil dispersion were incubated at the same conditions.

## Analyses

### Oil droplet size and concentrations

Concentrations and size distributions of oil droplets were determined by Coulter Counter measurements (Beckman Multisizer 4; Beckman Coulter Inc., Brea, CA, U.S.A), fitted with 280 μm aperture. This apparatus was used for measurements of droplets within a diameter range 5.6–100 μm. Sterile-filtered (0.22 μm) seawater was used as electrolyte in the Coulter Counter. Droplet concentrations were determined from volume concentrations (μm^3^/ml) and recalculated (mg/L) based on the density of the fresh oil.

### Chemical analyses

Alliquots of dispersed oil treatments dispersions were solvent-solvent extracted (dichloromethane, DCM) and analysed by gas chromatographic methods. Flasks were rinsed with DCM after removal of dispersions to extract oil attached to the glass walls. Extracts of dispersions and glass walls were pooled. Total extractable organic carbon (TEOC) was analysed by GC-FID, while quantification of 57 individual targeted compounds (*n*C10-*n*C36 n-alkanes, decalins, phenols, 2- to 5-ring polycyclic aromatic hydrocarbons (PAH) and 17α(H),21β(H)-Hopane) was performed by GC-MS analysis, as previously described [16]. In the GC-MS analysis, response values for individual target analytes were determined and based on a signal-to-noise ratio > 10. The lower limit of detections (LOD) varied from 0.001 to 0.01 μg/L for individual oil compounds. Target analytes were normalized against 17α(H),21β(H)-Hopane [17, 18] and sterile controls. Concentrations are reported as a percent of the initial oil concentration.

### DNA extraction and 16S rRNA gene data processing

Seawater samples (~ 500 ml, with and without oil and oil dispersions) were filtered through 0.22 μm filters (MilliporeSigma Durapore®, Merck, NJ, USA). Genomic DNA was extracted from the filters using a FastDNA Spin kit for soil (MP Biomedicals, CA, USA) according to the manufacturer’s instructions. Genomic DNA concentrations were quantified using Qubit 3.0 (ThermoFisher Scientific, MA, USA) with a dsDNA High Sensitivity kit (ThermoFisher Scientific, MA, USA).

Amplicons of 16S rRNA were generated according to Illumina’s “16S Metagenomic Sequencing Library Preparation” protocol using the S-D-bact-0341-b-S-17 and S-bact-0785-a-A-21 primer set [19]. Amplicons obtained by PCR were purified using magnetic beads (Agencourt Amoure XP Beads). Libraries were quantified using Quant iT Picogreen Dye and the Fragment Analyzer (Advanced Analytical) on Agilent’s Bioanalyzer. All amplicons were pooled equimolar and then sequenced paired-end on the Illumina MiSeq platform, 2 × 300 nt, following manufacturer instructions.

Raw pair-end reads were assembled with fastq-join using QIIME 1.9.1 [20]. Assembled sequences were demultiplexed and quality filtered to remove low quality reads (Phred score < 20; −q 19). UCHIME was employed for chimera detection on assembled quality filtered reads [21]. Operational taxonomic units (OTUs) were determined by clustering assembled sequences on 97% nucleotide identity using UCLUST [22] with open reference clustering option. Representative sequences were aligned with PyNAST [23] and taxonomy assignment was performed with RDP classifier [24] based on SILVA-123 database [19]. QIIME pipeline generated biome file was used as input for R Phyloseq package v.1.12.2 [25] for beta diversity analysis and generating community composition figures. Only sequences that had mean relative abundances (*n* = 3) > 1% of the total community relative abundance, at least at one time point, were included in the graphical output for the community composition.

### Multivariate data analysis

Total microbial community data were agglomerated to the family level, replicates were averaged and relative abundances were calculated with the Phyloseq package [25]. The chemistry data were normalized to 17α(H),21β(H)-Hopane and compared to the sterile controls to describe the degradation of each component as a percentage of the starting concentration at each timepoint in the absence of abiotic losses.

The time series for each oil and temperature contained different sampling points (Fig. 1). To obtain identical time points for better comparison, a local polynomial regression was fitted to the data for each individual (each component/species for each temperature and each oil) time series using the *loess* function in the R stats-package (v.3.2.2). The resulting line fit (loess-model) was used to interpolate the % loss of oil components and the relative abundance of bacterial families at days 0, 3, 6, 9, 13, 16, 21 and 30. For the modelled data, the minimum value was set to zero to account for any sub-zero predictions. For the microbial data, this was achieved by normalizing the data to unity (sum all abundances = 1). The modelled time series were then used for PCA analysis and for describing compositional changes over time.

For total microbial community data, PCA was performed using sparse PCA from the mixOmics-package (*spca*, on centered and scaled data using 3 components) [26]. For the chemistry data, *prcomp* (centered and scaled) from the base stats package was used. After generating the PCA models, the scores were extracted, and plots were generated with *ggplot2*. For the modelled microbial data, a smoothed *loess* function was added to the basic point-plot (geom_point) using stat_smooth for each sequential group of four time points. For the modelled chemistry data, a single smoothed function was added which was based on all four time series using geom_smooth defaults (0.95 confidence interval, loess function). Regressions using *pls* were built via *Clustered Image Map* (CIM) function (within mixOmics package) with chemistry data as xblock and microbial community data as yblock to study correlations and clustering between datasets.

## Results

### Chemical analyses

**Fig. 2:**
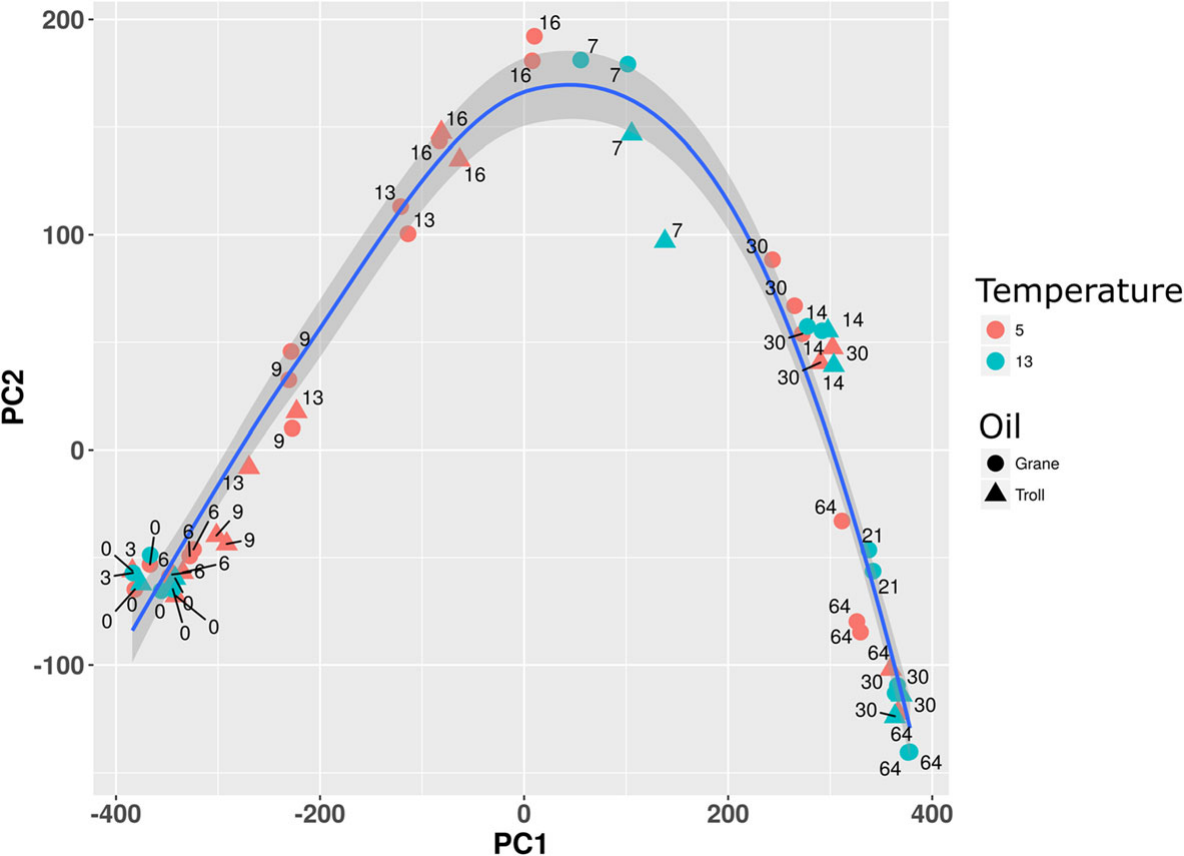
PCA of the chemical profile of chemically dispersed Grane and Troll oil incubated with seawater at 5 °C and 13 °C. Each flask is represented as either a circle (Grane oil) or a triangle (Troll oil), and each symbol represents the composition of 57 individual targeted compounds normalized to hopane at that specific time point. Modelled data are present in the figure alongside experimental data to statically analyse the complete data set. Red symbols indicate an incubation temperature of 5 °C and blue symbols indicate an incubation temperature of 13 °C. 83.6 and 8.3% of variations are explained with PC1 and PC2, respectively

The chemistry dataset gathered in this study characterized 57 oil components from two oils at two temperatures over 64 days with different sampling time points (Fig. 1). Biodegradation half-lives are presented in Additional file 2: Table S2. A PCA score plot was generated to condense the complex data and enable analysis among oil types and temperatures. The PCA plot of the chemical profiles (GC-MS and GC-FID results) within individual treatment replicates illustrated a similar trajectory among oil types and temperatures, but clustering of experimental replicates incubated with different oils and different temperatures revealed several trends in alkanes and aromatic biodegradation among the four experiments (Fig. 2). Patterns of biodegradation can be compared among treatments, as the PAH/alkane ratio is represented in PC2 and total oil biodegradation represented in PC1. As time points increased along the PC2 axis, alkane biodegradation surpassed PAH biodegradation and the slope of the curve became positive. The top of the bell curve (the PC2 maximum) illustrates when the loss of alkanes and PAHs was most similar. As the experiments proceeded, alkanes were depleted, and the loss of PAHs exceed that of alkanes and thus the slope of the curve becomes negative. In Grane and Troll incubations at 13 °C, the largest separation on the PC1 axis was observed between day 0–6 and day 7, with day 0–6 incubations all clustering together (Fig. 2). Another strong cluster between Grane and Troll incubations formed after the PC2 maximum, where day 30 (5 °C) and 14 (13 °C) were found in close proximity (Fig. 2).

When the composition of individual hydrocarbons were compared between the two oils at the same time point, the two oils grouped separately at 5 °C but not 13 °C (Fig. 2). The biodegradation of Grane at day 6 (5 °C and 13 °C) clustered with the biodegradation of Troll at day 9 (5 °C), while day 9 (Grane oil at 5 °C) and day 13 (Troll oil at 5 °C) clustered together. Further along the PC1 axis, day 13 (Grane oil at 5 °C) and day 16 (Troll oil at 5 °C) clustered together (Fig. 2). Day 16 (dispersed Grane oil at 5 °C) and day 7 (dispersed Grane and Troll at 13 °C) dominated the top of the bell curve, where the slope of the line approaches 1 and changes among alkanes and PAHs are most similar. After the apex, day 30 (both oils at 5 °C) was grouped with day 14 (both oils at 13 °C) (Fig. 2).

### Microbial community analysis

**Fig. 3:**
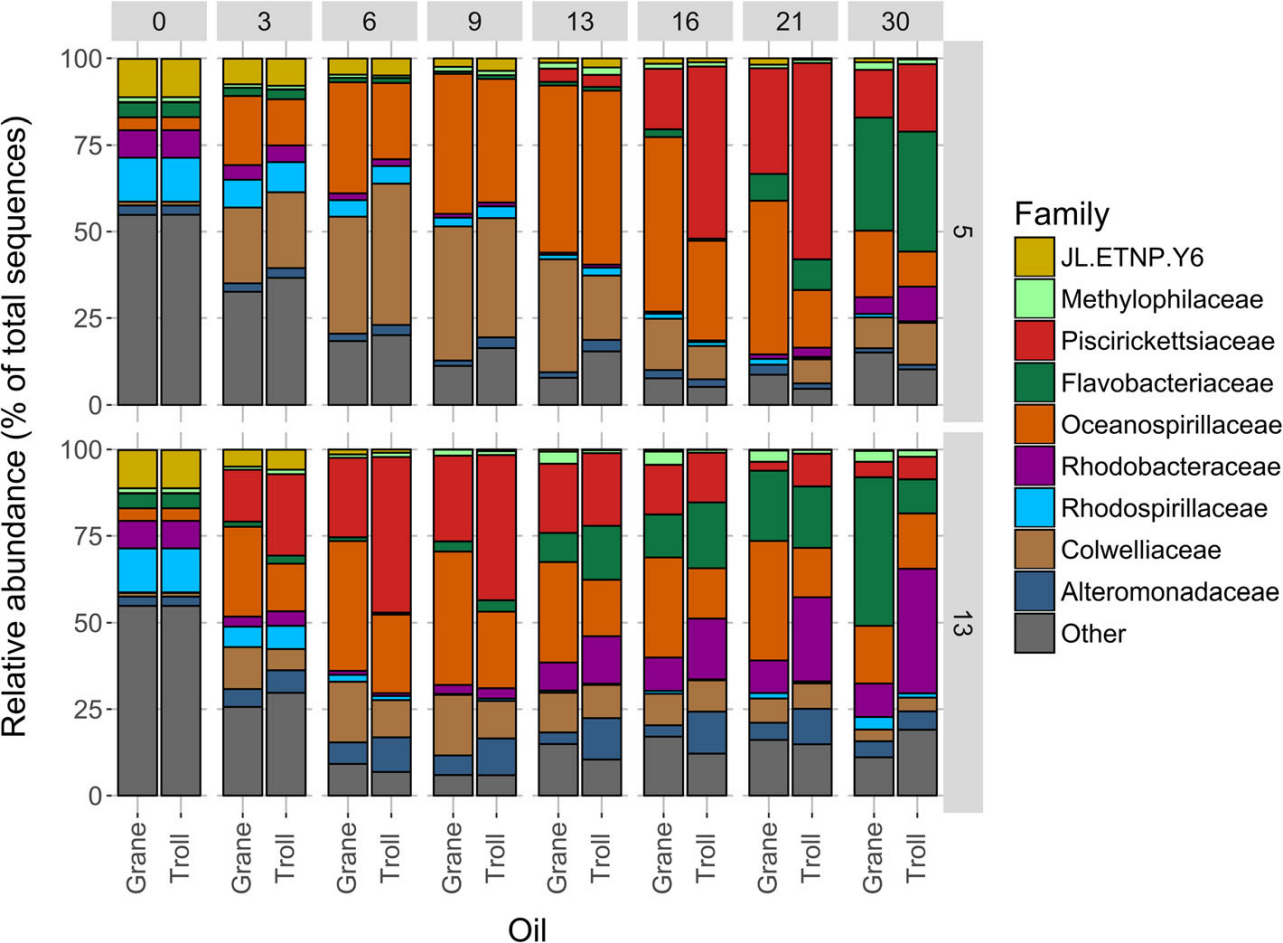
Mean relative abundance of bacterial families in each treatment from modelled data. Flaks contained seawater incubated with chemically dispersed Grane and Troll oil at 13 and 5 °C (2–3 mg/L; DOR 1:100). Figure includes abundances that were > 1% of the total community

Modeled timeseries data can predict microbial community structure in Troll and Grane oil dispersions at the different temperatures. The main difference in microbial community structure between 5 °C and 13 °C emerged from the relative abundance of the *Colwelliaceae* family. At the lower temperature (5 °C), *Colwelliaceae* increased in abundance at day 3 in both oils (by 22%) compared to the unamended control. After day 6, *Colwelliaceae* exhibited consistently higher abundance in Grane incubations compared to Troll at both temperatures. The relative abundance of *Colwelliaceae* was slightly greater in Troll dispersions than Grane dispersions at day 6 (41% vs. 34%), but by day 9 there was more *Colwelliaceae* in the Grane incubation than the Troll (39% vs. 35%) (Fig. 3). By the day 30, abundance of *Colwelliaceae* decreased substantially (to ~ 10% of the total community). At 13 °C, response of *Colwelliaceae* were rather modest and exhibited maximal values at days 6 and 9 (17.6, 10.8 and 17.5%, 10.9% for Grane and Troll, respectively). *Colwelliaceae* were also prominent at 5 °C control incubations compared to 13 °C, where after 9 days abundance reached 26.9% and was maintained until end of experiment (unlike in oil incubations) (Additional file 4: Figure S1). At 13 °C, *Colwelliaceae* showed rather modest increase in abundance in control incubations (< 10%), except the sample from incubation day 7 (33.5%) (Additional file 4: Figure S1).

*Oceanospirillaceae* responded to both oil dispersions at both temperatures by day 3 (Fig. 3). The relative abundance of *Oceanospirillaceae* peaked at day 9 in the13 °C incubation (38.4 and 22.1% for Grane and Troll, respectively), and peaked at day 13 in the 5 °C incubation with Troll (50%) and at day16 in the 5 °C incubation with Grane (51%) (Fig. 3). *Oceanospirillaceae* seemed to favor Grane to Troll oil at both temperatures and had higher abundances at 5 °C compared to 13 °C (Fig. 3). *Oceanospirillaceae* were also abundant also in controls, with higher abundances at 5 °C than 13 °C, but these abundances were low compared to oiled incubations (Additional file 4: Figure S1).

Throughout the incubation, *Piscirikettsiaceae* and *Flavobacteriaceae* exhibited different patterns of relative abundance between the two temperatures. The increase in the relative abundance was earlier at 13 °C than 5 °C for both families (Fig. 3). It was observed that *Piscirickettsiaceae* preferred dispersed Troll oil over Grane at both temperatures, and that somewhat higher abundances were achieved at lower temperatures. *Piscirickettsiaceae* were not observed in control samples. The highest relative abundance of *Flavobacteriaceae* occurred at 13 °C, with more *Flavobacteriaceae* responding to dispersed Grane oil (43%) than Troll (10%) at day 30. At 5 °C the *Flavobacteriaceae* only responded after 21 days, and relative abundances reached maximum values at day 30 in both dispersed Grane oil (33%) and Troll oil (35%) (Fig. 3). Preferences of *Flavobacteriaceae* towards a certain oil type or temperature are rather inconclusive based solely on abundances over the incubation period. *Flavobacteriaceae* also increased in control incubations for both temperatures, but only towards the end of experiment (day 64, > 15%), otherwise abundances were < 5% (Additional file 4: Figure S1).

*Alteromonadaceae* and *Rhodobacteraceae* were more dominant at 13 °C than 5 °C and seemed to prefer Troll oil over Grane oil at the higher temperature (Fig. 3). The abundance of *Alteromonadaceae* at 5 °C did not exceed 3.5% for any of the oil, but at 13 °C *Alteromonadaceae* reached its highest relative abundance between day 13–16 with 12%. At 13 °C, *Rhodobacteraceae* decreased in relative abundance between day 3–9 compared to day 0; however, at day 13 *Rhodobacteraceae* started to increase and reached its highest abundance on day 30 in both oil incubations (36% in Troll and 10% in Grane). *Alteromonadaceae* were not detected in control samples (where the cut-off of 3% in median abundance was applied), while *Rhodobacteraceae* maintained the abundance throughout control incubation (< 10%) (Additional file 5: Figure S2).

**Fig. 4:**
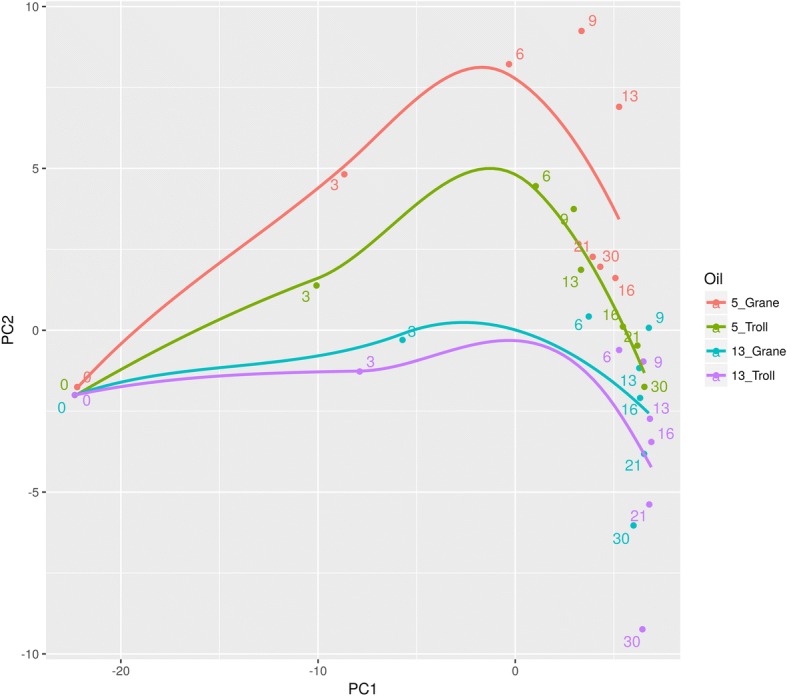
PCA of the mean relative abundance of bacterial families in each treatment. Flaks contained seawater incubated with chemically dispersed Grane and Troll oil at 13 and 5 °C (2–3 mg/L; DOR 1:100). Each treatment is represented as a different colour and the legend details the incubation temperature followed by the oil type. Modelled data are present in the figure alongside experimental data to statically analyse the complete data set. Numbers indicate incubation days. PC1 and PC2 explain 58.2 and 9.5% of variations, respectively

To identify shifts within the total microbial community in the absence of abundance cut-offs, we performed a PCA analysis of the total sequence data. Patterns along the PC2 axis revealed the influence of temperature on microbial community dynamics, as incubations conducted at 5 °C grouped above zero, and 13 °C incubations grouped below (Fig. 4). Microbial community structures in Grane and Troll dispersions were more similar to one another at 13 °C than at 5 °C (Fig. 4). From the loadings (Additional file 6: Figure S3), we observed that the major differences of the most important players contribute for different PC2 loadings.

### PLS2 analysis of chemistry and microbial community data

**Fig. 5:**
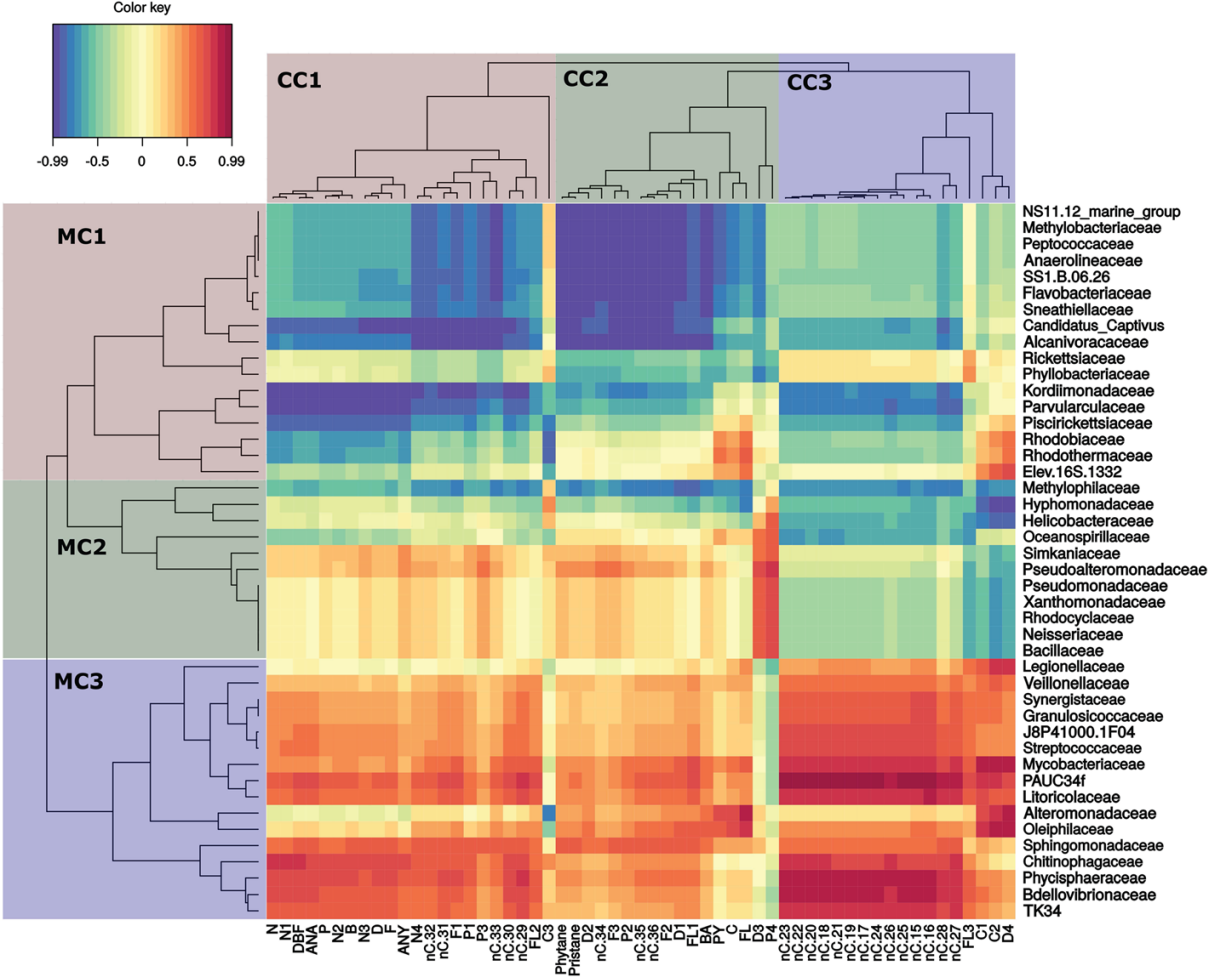
Grane oil at 5 °C. Heatmap clustering of individual target hydrocarbons based on modelled degradation curves and the mean relative abundance of microbial families. Red color indicates a strong positive correlation between hydrocarbon persistence and bacterial family and thus suggests that biodegradation is not corellated to that family. Blue indicates a strong negative correlation between persistence and bacterial family and thus suggests that biodegradation is corellated to that family. On the left, microbial clusters (MC) are grouped by color and identified as MC1, MC2, or MC3. Single compound clusters (CC) are also grouped by color and identified as CC1, CC2, and CC3. In single compound clusters (CC) n-alkanes are abbreviated by nC. acronym, followed by the number of carbon atoms describing the chain length. Abbreviations for aromatic compounds are further described in additional table (Additional file 3: Table S3)

**Fig. 6:**
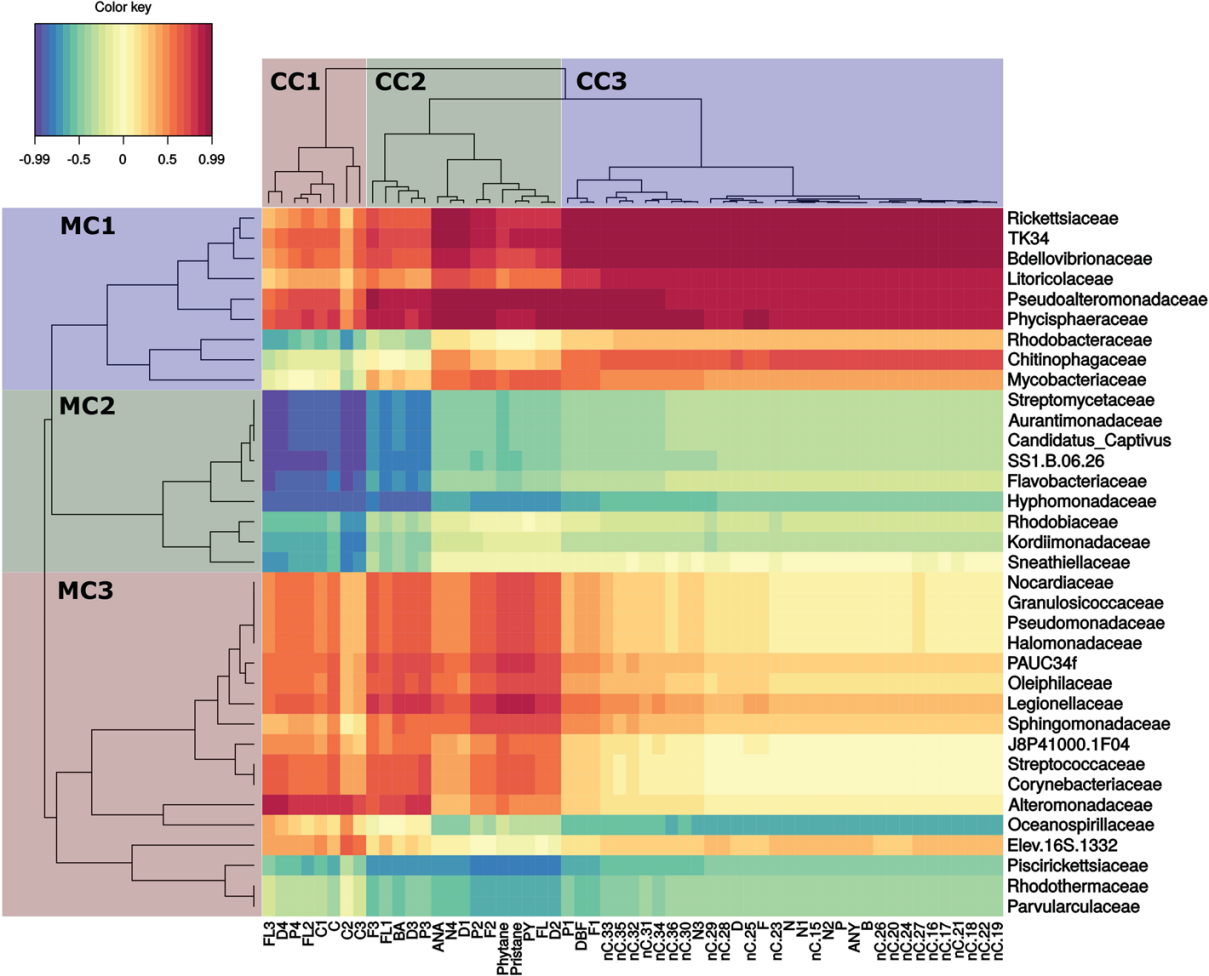
Troll oil at 5 °C. Heatmap clustering of individual target hydrocarbons based on modelled degradation curves and the mean relative abundance of microbial families. Red color indicates a strong positive correlation between hydrocarbon persistence and bacterial family and thus suggests that biodegradation is not corellated to that family. Blue indicates a strong negative correlation correlation between hydrocarbon persistence and bacterial family and thus suggests that biodegradation is corellated to that family. On the left, microbial clusters (MC) are grouped by color and identified as MC1, MC2, or MC3. Single compound clusters (CC) are also grouped by color and identified as CC1, CC2, and CC3. In single compound clusters (CC) n-alkanes are abbreviated by nC. acronym, followed by the number of carbon atoms describing the chain length. Abbreviations for aromatic compounds are further described in additional table (Additional file 3: Table S3)

**Fig. 7:**
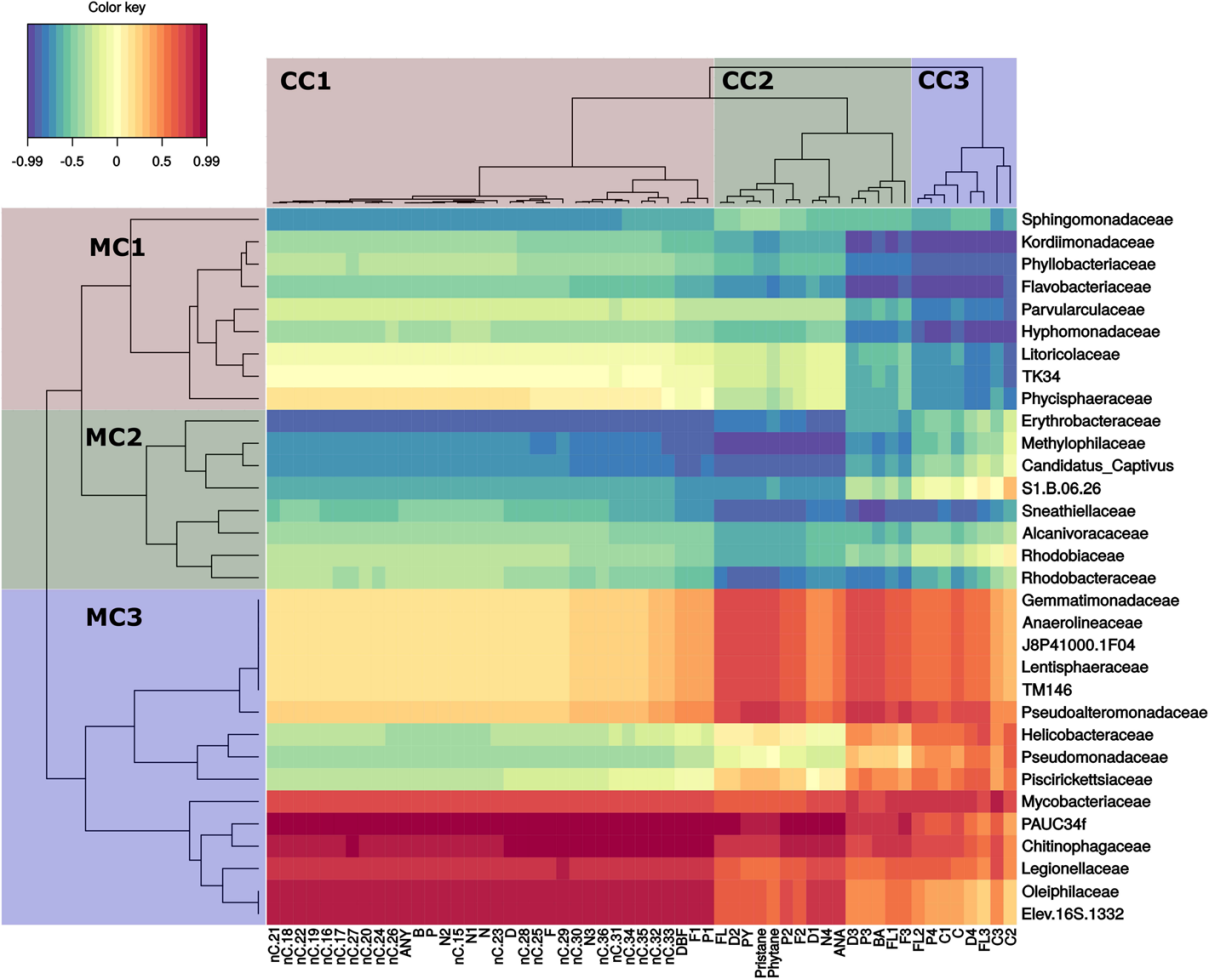
Grane oil at 13 °C. Heatmap clustering of individual target hydrocarbons based on modelled degradation curves and the mean relative abundance of microbial families. Red color indicates a strong positive correlation between hydrocarbon persistence and bacterial family and thus suggests that biodegradation is not corellated to that family. Blue indicates a strong negative correlation between hydrocarbon persistence and bacterial family and thus suggests that biodegradation is corellated to that family. On the left, microbial clusters (MC) are grouped by color and identified as MC1, MC2, or MC3. Single compound clusters (CC) are also grouped by color and identified as CC1, CC2, and CC3. In single compound clusters (CC) n-alkanes are abbreviated by nC. acronym, followed by the number of carbon atoms describing the chain length. Abbreviations for aromatic compounds are further described in additional table (Additional file 3: Table S3)

**Fig. 8:**
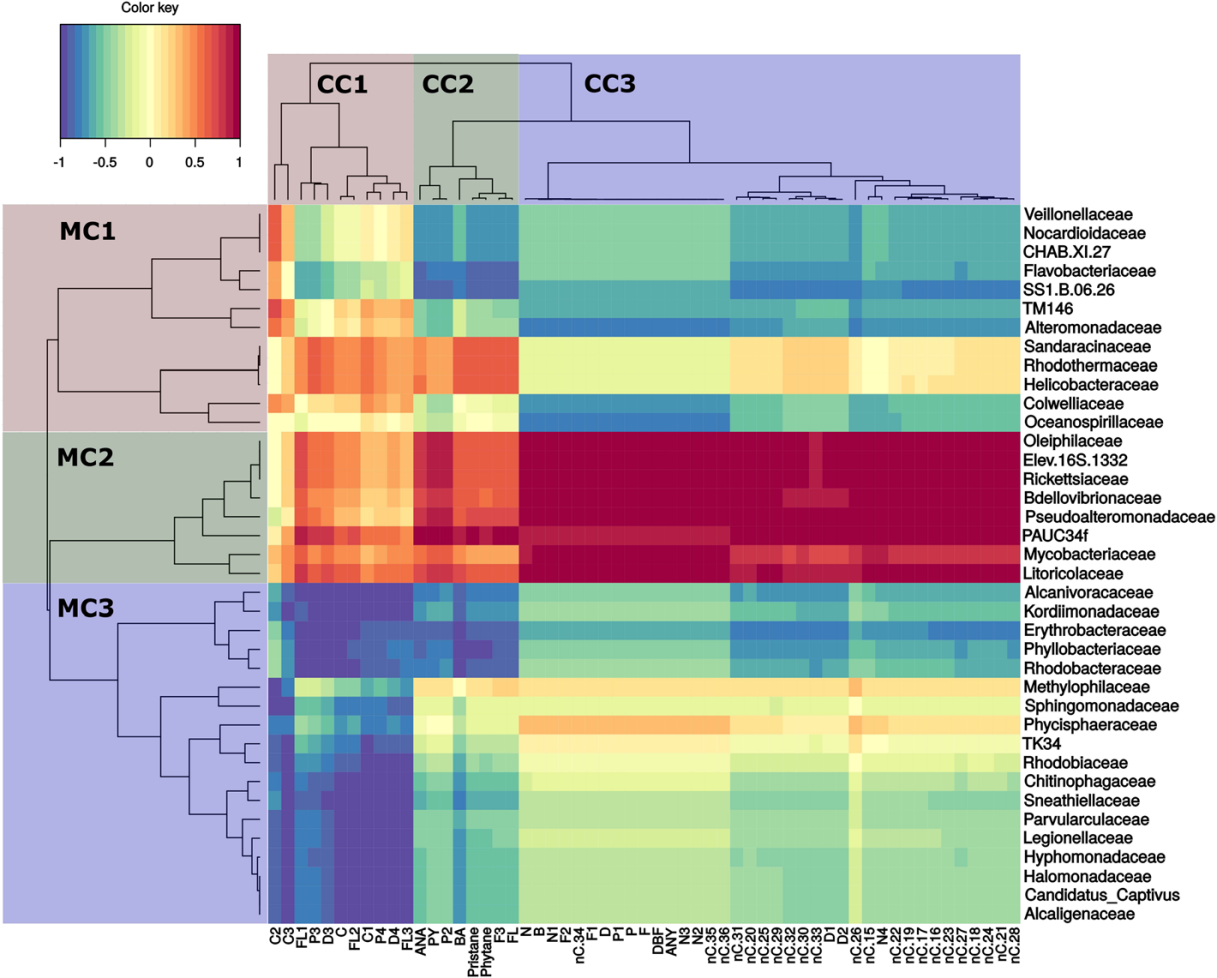
Troll oil at 13 °C. Heatmap clustering of individual target compounds based on modelled degradation curves and the mean relative abundance microbial families. Red color indicates a strong positive correlation between hydrocarbon persistence and bacterial family and thus suggests that biodegradation is not corellated to that family. Blue indicates a strong negative correlation between hydrocarbon persistence and bacterial family and thus suggests that biodegradation is corellated to that family. On the left, microbial clusters (MC) are grouped by color and identified as MC1, MC2, or MC3. Single compound clusters (CC) are also grouped by color and identified as CC1, CC2, and CC3. In single compound clusters (CC) n-alkanes are abbreviated by nC. acronym, followed by the number of carbon atoms describing the chain length. Abbreviations for aromatic compounds are further described in additional table (Additional file 3: Table S3)

Correlations between the presence of individual oil compounds and the relative abundance of bacterial families were conducted using multivariate approaches of the two modelled datasets. The results are presented in heatmaps (Figs. 5, 6, 7, 8) and described below.

### Biodegradation of dispersed Grane oil at 5 °C

Biodegradation curves from modelled data and microbial families formed three distinct clusters (Fig. 5). Chemical cluster 1 (CC1) contained parent and alkylated 2- to 3-ring aromatics, long-chain *n*-alkanes, and naphthalenes (C-C4). The persistence of CC1 compounds correlated negatively (hereinafter “well correlated”) with cluster 1 of microbial community (MC1) and indicates a strong correlation between biodegradation and the identified bacterial families. CC2 consisted of complex polyalkylated 2- to 3-ring and 4- to 6-ring PAHs and larger n-alkanes, but also isoprenoids. The biodegradation of CC2 compounds also correlated well with MC 1 (blue color, Fig. 5). CC3 consisted of a few 2- to 3-ring and 4- to 6-ring PAHs and mostly middle-chain *n*-alkanes and its biodegradation correlated most strongly with the MC1 and MC2 (blue color, Fig. 5).

Nine bacterial families from MC1 formed a tight subcluster within the main MC1 (NS11.12_marine_group to *Alcanivoracaceae*) and they all had strong correlations to the biodegradation of complex polyalkylated 2- to 3-ring and 4- to 6-ring PAHs and *n*C34+ alkanes within CC2 (Fig. 5). To a lesser extent, this subgroup was also correlated to the biodegradation of isoprenoids and *n*C29-*n*C33 alkanes within CC1 (Fig. 5). *Kordiimonadaceae*, *Parvularculaceae* and *Piscirikettsiaceae* from MC1 showed affinity towards CC1 (*R* < − 0.7), in particular naphthalenes, dibenzothiophenes and *n*C29-*n*C33 alkanes. In addition, *Kordiimonadaceae* and *Parvularculaceae* correlated strongly with *n*C15- *n*C28 alkanes (R < − 0.7) from the CC3. Within MC2, *Oceanospirillaceae*, *Helicobacteraceae*, *Hypomonadaceae* and *Methylophylaceae* correlated (*R* < − 0.65) with *n*C15-*n*C28 from CC3, whereas *Methylophylaceae* showed also correlation with CC1 and CC2 (Fig. 5).

### Biodegradation of dispersed troll oil at 5 °C

Modelled biodegradation curves and the relative abundance of microbial families also formed three distinct clusters in incubations with Troll oil at 5 °C (Fig. 6). Among the three clusters of degradation curve profiles, CC1 was the tightest cluster and contained 8 complex C2+ alkylated (C2 and greater) 2- to 3-ring PAH and 4- to 6-ring PAHs and correlated well with MC2 (*R* < − 0.7). Representatives within MC2 were *Kordiimonadaceae, Rhodobiaceae, Hypomonadaceae, Oceanospirillales* strain SS1.B.06.26, *Candidatus Captivus*, *Aurantimonadaceae* and *Streptomycetaceae.* CC2 consisted of mostly of 2- to 3-ring PAHs and branched alkanes and correlated well with prokaryotes from MC2 and with *Piscirikettsiaceae*, *Rhodothermaceae* and *Parvulaculaceae* from MC3. Interestingly, most of dibenzothiophenes (C1-C3) were located within this CC2 cluster. Finally, CC3 consists of the whole spectrum of *n*-alkanes and most of naphthalenes (C-C3) and few other 2- to 3-ring PAHs. This cluster correlated well with *Oceanospirillaceae* (R < − 0.65).

### Biodegradation of dispersed Grane oil at 13 °C

Similar to the other treatments, the biodegradation curves for Grane oil at 13 °C were also arranged in three clusters (Fig. 7). The first cluster (CC1) contains the whole *n*-alkane spectrum, naphthalenes (C-C3) and the 2- to 3-ring PAHs (parent and C1 alkylated). CC1 correlated well (R < − 0.65) with MC2, with the most prominent families being *Erythrobacteraceae*, *Methylophilaceae*, *Candidatus Captivus* and family SS1.B.06.26. *Sphingomonadaceae* was the only family in MC1 that exhibited a strong correlation to CC1. CC2 contained C2+ alkylated 2- to 3-ring PAHs and a few 4- to 6-ring PAHs and isoprenoids. CC2 had good correlation (*R* < − 0.7) with most representatives from MC2, but also with *Flavobacteriaceae* and *Kordiimonadaceae* from MC1 (Fig. 7). CC3 consisted of mostly polyalkylated 4- to 6-ring PAHs and correlated (R < − 0.7) with *Kordiimonadaceae, Phyllobacteriaceae, Flavobacteriaceae, Parvularculaceae, Hypomonadaceae* and *Litoricolaceae* from MC1, and *Sneathiellaceae* and *Rhodobacteraceae* from MC2 (Fig. 7).

### Biodegradation of dispersed troll oil at 13 °C

Similar to the Troll oil at 5 °C and the Grane at both temperatures (13 °C and 5 °C), three chemical clusters and three microbial clusters were also present in the heatmap for Troll oil at 13 °C. C4 alkylated 2- to 3-ring PAHs and polyalkylated 4- to 6-ring PAHs made up CC1, which correlated well with most families from MC3 (R < − 0.7) (Fig. 8). CC2 consisted of 2- to 3-ring PAHs and branched alkanes and aligned with a subcluster of MC3 (*Alcanivoracaceae* to *Rhodobacteracea*e) and MC1 (*Veillonellacae* to *Oceanospirillales* SS1.B.06.26)) (*R* < 0.7). The third chemical cluster (CC3) consisted of the whole spectrum of naphthalenes (C-C4), C-C2 dibenzothiophenes and the whole spectrum of *n*-alkanes. Within MC1, *Alteromonadaceae* was mostly correlated to the entire CC3, while *Flavobacteriaceae* and *Oceanospirillales* strain SS1.B06.26 had a stronger correlation to the alkanes and *Colwelliaceae* and *Oceanospirillaceae* had a stronger correlation to the naphthalenes (C-C4), C-C2 dibenzothiophenes. In addition, a small subcluster of MC3 (*Erythrobacteraceae*, *Phyllobacteriaceae* and *Rhodobacteraceae*) also exhibited correlation to the alkanes in CC2.

## Differences between oils and temperatures

### Chemical single compound clustering

Four different heatmaps illustrate similarities in biodegradation through the clustering of individual hydrocarbons based on the similarity of slopes of their biodegradation (i.e. decay) curves. In both oils and at both temperatures, the majority of *n*-alkanes and naphthalenes clustered together and clustered apart from 2- to 3-ring and 4- to 6-ring PAHs. This clustering was not uniform and varied between oils and temperatures and was dependent on alkylation rate as well as hydrocarbon chain length. Clustering among alkanes exhibited minor differences, but the majority of alkanes clustered in a similar fashion among oil types and temperatures. In the Grane incubation at low temperature (5 °C), higher molecular weight *n*-alkanes were found in CC1 and CC2, but the majority of *n*-alkanes were found within CC3 (Fig. 5). In contrast, all alkanes were present in one distinct cluster (CC3) in the Troll incubations at 5 °C (Fig. 6) and 13 °C (Fig. 8), as well as the Grane incubation at 13 °C (Fig. 7). Surprisingly, in Grane incubations at 5 °C, the degradation rates of 4- to 6-ring PAHs (CC2) were more similar to *n*-alkanes (CC3) than the degradation rates of 2- to 3-ring PAHs (CC1) (Fig. 5).

The biodegradation of dibenzothiophene was observed for Troll oil, but not for Grane. The chemical formulation of Grane (i.e. low concentration of dibenzothiophene) prevented dibenzothiophene’s biodegradation curve to be determined. In seawater incubations with Troll oil at 5 °C, dibenzothiophene was clustered with the 2- to 3-ring PAHs, but at 13 °C dibenzothiophenes clustered with naphthalenes and *n*-alkanes.

### Clustering of microbial families

Heatmaps did not reveal consistent clustering of microbial families between the oils and temperatures (Figs. 5, 6, 7 and 8). Although, MC1 and MC2 in the incubation with Grane at 13 °C (Fig. 7) resembled MC3 in the Troll incubation at 13 °C (Fig. 8) and these clusters were correlated to similar chemical compounds. To some extent, patterns between the two Grane incubations can be seen as MC1 and MC2 contained similar bacterial families at the two different temperatures (Figs. 5 and 7). Nevertheless, there is no clear pattern in clustering of microbial families among oils and temperatures, confirming the differences shown by the PCA output based on microbial community composition (Fig. 4). Overall, *Alcanivoracaceae, Candidatus Captivus, Erythrobacteraceae, Flavobacteriaceae, Hyphomonadaceae, Oceanospirillaceae, Rhodobacteraceae,* and *Oceanospirillales* strain SS1.B.06.26 consistently dominated the microbial community, suggesting these families may be involved in biotransformation of dispersed oil in our incubations.

## Discussion

In this study, we investigated a multivariate approach for the combined analysis of chemistry and microbial data to study the effects of temperature and oil composition on the biodegradation of chemically dispersed oil. Heatmap clustering of modelled microbial community composition did not produce consisting clustering of microbial taxonomic families between the two temperatures and oil types; however, we did observe trends in the relative abundance of specific taxonomic families at different temperatures and oil types. *Alcanivoracaceae, Candidatus Captivus, Erythrobacteraceae, Flavobacteriaceae, Hyphomonadaceae, Oceanospirillaceae, Rhodobacteraceae,* and *Oceanospirillales* strain SS1.B.06.26 consistently dominated the microbial community structure, suggesting these families may be involved in the biodegradation of chemically dispersed oil. Clustering of modelled biodegradation curves reveled a strong separation between different oils at the same temperature, indicating that oil composition has a strong effect on biodegradation rates. As expected, temperature produced a substantial effect on the biodegradation pattern of individual alkanes and aromatics, with losses at 13 °C occurring approximately twice as fast as those at 5 °C. Overall, we found that our modelled chemistry data (Figs. 2, 5, 6, 7 and 8) followed the same trends as our calculated biodegradation rates (Additional file 2: Table S2) and allowed comparison of experiments sampled at different time points.

### Biotransformation of dispersed oil

Soluble low molecular weight compounds (BTEX, phenols, naphthalenes) are usually associated with water accommodated fractions (WAF), while *n*C6+ saturates and high molecular weight PAHs are commonly associated with particulate oil. During oil biodegradation, soluble hydrocarbons are generally utilized faster than compounds in the oil phase, as they are easily transported from the water phase into the cell [27]. Interestingly, biodegradation rates of low soluble alkanes have been shown to exceed their respective solubility rates [28], which indicates that oil-degrading bacteria are capable of degrading alkanes in particulate oil. While the bioavailability of PAHs is dependent on solubility, the bioavailability of saturates is mainly influenced by physicochemical characteristics of the oil [29].

The PCA plot of chemical profiles compared biodegradation patterns (i.e. PAH/alkane ratio) among the four different incubations and illustrated a similar trajectory among oil types and temperatures (Fig. 2). In addition, when biodegradation patterns at the same time point were compared between incubations, Troll and Grane grouped together at 13 °C but not at 5 °C; suggesting that biodegradation patterns are more dependent upon temperature than oil type. Low temperatures are known to influence the bioavailability of petroleum hydrocarbons [30], which in turn can affect the type and amount of biodegradation genes expressed by oil-degrading microorganisms. Biodegradation genes were not identified in our incubations, but it is likely that similar biodegradation genes and pathways were expressed [31, 32], with gene expression delayed at the colder temperature. Therefore, oil type and temperature are likely to affect the onset of specific biodegradation pathways by impacting the amount of soluble petroleum hydrocarbons available to oil-degrading microbes.

For both oils, faster transformation rates were observed for *n*-alkanes and aromatics at the higher temperature (Fig. 2; Additional file 1: Table S1). In comparing Troll to Grane at 5 °C, tighter clustering of *n*-alkanes (*n*C15 – *n*C36) in Troll was observed (Figs. 5 and 6) and the majority of HMW *n*-alkanes (*n*C31 – *n*C36) had longer half-lives in the Grane incubation (Additional file 1: Table S1). In addition, the more alkylated PAHs also had longer half-lives in the Grane incubation (Additional file 1: Table S1). These results suggest that the bioavailability of HMW saturates and alkylated aromatics were more susceptible to temperature in incubations with Grane oil than Troll. This is supported by the physical characteristics of the two oils. Since Grane is a highly viscous asphaltic crude (Table 1), the bioavailability of compounds associated with particulate oil would be more limited at lower temperatures compared to a lighter oil (e.g. Troll). Surprisingly, alkanes ranging from *n*C15 – *n*C30 had higher biodegradation half-lives and thus slower biodegradation rates in Troll oil than the more viscous Grane oil at 5 °C (Additional file 1: Table S1). It is likely that the larger fraction of the light aromatic hydrocarbons of Troll incubation was inhibiting biodegradation. This phenomenon was already observed by RM Atlas [2], where he noticed that light crudes became less degradable at low temperatures compared to heavy crudes. On the other hand, it seems that the bioavailability of long chain *n*-alkanes (nC29+) was decreased in Grane oil compared to Troll based on oil viscosity. This trend in the biotransformation of *n*-alkanes was also observed in clustering, as the decay curves of long chain *n-*alkanes clustered differently between the two oil types at 5 °C (Figs. 5 and 6) and oils grouped separately before reaching the apex in the bell-shaped curve (Fig. 2).

In addition to temperature dependence in oil biodegradation experiments, clustering based on oil-type and the biodegradation of individual compounds should also be implemented when modelling the fate of oil. For example, the Oil Spill Contingency And Response (OSCAR) model groups compounds based on molecular weight and boiling points, which may overlook the biodegradation of individual compounds. Therefore, there is need to refine such an approach, as compounds may not follow biotransformation patterns based solely on these conditions.

### Microbial community dynamics

Modelled microbial community datasets revealed differences between the two temperatures and oils, especially at low temperature. Throughout the incubation, microbial community structure in Troll and Grane incubations appeared more similar at 13 °C than at 5 °C. At the end of the incubation, the microbial community in incubations with Troll at 5 °C was more similar to communities present at 13 °C in both Troll and Grane incubations than to communities exposed to Grane oil at 5 °C (Fig. 4). Principal oil-degraders observed in this study are well known hydrocarbon biodegrading microbes observed in a variety of studies following the Deepwater Horizon (DWH) spill. *Oceanospirillaceae*, which was one of the initial families to increase in our incubations at both temperatures, was also detected in the DWH as a principal microorganism for *n*-alkane degradation [33, 34]. *Colwelliaceae*, on the other hand, was associated with the biodegradation of both *n*-alkanes and aromatics [33, 35]. In both oils, *Colwelliaceae and Oceanospirillaceae* were more prominent in colder (5 °C) than warmer (13 °C) incubation (Fig. 3). *Oleispira* (a genus of *Oceanospirillaceae*) and *Colwellia* (a genus of *Colwelliaceae*) are commonly found in Arctic environments in response to oil [36, 37]. *Piscirickettsiaceae* were active in our study at both temperatures, preferring Troll oil to Grane oil, and were also enriched in the deepsea plume of dispersed oil during the DWH [33]. *Flavobacteriaceae*, *Rhodobacteraceae* and *Alteromonadaceae* also increased in abundance during the DWH after *Oceanospirillaceae* and *Colwelliaceae* peaked, suggesting that members of these microbial families may consume biodegradation metabolites [33, 38, 39].

The approach of presenting the most abundant taxa is a common practice as they are usually the most influential; however, highly abundant taxa may overshadow the role of low abundant species in biodegradation. Biodegradation occurs throughout the community and rare taxa may be more important than previously thought [40]. By correlating the modelled data in cluster heatmaps (Figs. 5, 6, 7 and 8), we were able to screen for individual microbes that may have played a key role in the loss of individual hydrocarbons. Some bacterial families correlated (*R* < − 0,65) with the loss of targeted analytes and were also found in literature to be involved directly or indirectly in biodegradation processes of hydrocarbons. *Flavobacteriaceae*, *Oceanospirillaceae, Piscirickettsiaceae* and *Rhodobacteraceae* include known oil-degrading microbes and were identified as the most abundant families in our incubations, but other taxa known to include oil-degrading microbes were also found in our incubations, but at a low abundance (< 1%) (e.g. *Alcanivoracaceae, Methylophilaceae, Sphingomonadaceae* and *Erythrobacteraceae*) [41–44].

Our data show that oil-degraders do not correlate perfectly with the biodegradation of individual hydrocarbons (e.g., *Oceanospirillaceae* in Grane at 13 °C, or *Piscirickettsiaceae* in Troll and Grane at 13 °C). One of the reason for this discrepancy may be that microbes need to increase in abundance prior to significant depletion of hydrocompounds. Therefore, a natural lag may exist between increases in relative abundances of specific taxa and the chemical loss of individual compounds. In addition, since oil degrading genes are so ubiquitous, there are likely many other taxa that are contributing to oil loss than just the most abundant microbes. Hence, it is important to note that the correlation does not imply causality, and drawing conclusions only based on correlation outcomes should be considered a poor practice.

Given the relatively few data points collected in this study, a full-scale time series analysis of the data was not pursued. Future studies should pursue analyses at a higher time-resolution, as this would expand the options for time-series analysis tools for microbial data. These possibilities have been excellently summarized by K Faust, L Lahti, D Gonze, WM de Vos and J Raes [45], where most methods are judged carefully as to not infer causal connections.

## Conclusions

In the current paper, our aim was to introduce a different approach for evaluating and presenting complex microbial and chemical datasets for oil biodegradation incubation experiments. As opposed to multiple univariate analysis usually applied to microbial and chemical analyses, we present here a multivariate approach of connecting microbial community and chemical datasets. Using samples obtained from different incubations at different time points, this multivariate approach allowed us to evaluate how different temperatures and oil types influence modelled biodegradation processes, including single compound depletion and changes in microbial community composition at specific time points. As expected, temperature was shown to have a strong effect on biodegradation efficiency and community dynamics. These results suggest that different oil types may influence degradation rates and microbial community structure, especially at low temperatures (5 °C). Finally, correlation of microbial taxa with individual hydrocarbons revealed the potential influence of low-abundant taxa on oil biodegradation in marine environments.

## Additional file

Additional file 1: **Table S1.** Experimental design. Sampling days and number of replicates are described for all experimental analyses. The following coding system is applied: X + Y + Z, where X is number of replicates for oil dispersions in natural unfiltered seawater, Y is number of replicates for dispersions in sterilized filtered seawater, and Z is number of replicates for natural unfiltered seawater without oil. (PDF 391 kb)

Additional file 2: **Table S2.** Calculated half-lives for targeted oil compounds. (PDF 72 kb)

Additional file 3: **Table S3.** List of abbreviations for aromatics compounds used in correlation plots. (PDF 40 kb)

Additional file 4: **Figure S1.** Microbial community composition in control samples over the incubation period. (PDF 103 kb)

Additional file 5: **Figure S2.** Biotransformation of targeted semivolatile n-alkanes (A and B) and PAH (C and D) in dispersions of Troll and Grane oils at 13 °C and 5 °C. (PDF 195 kb)

Additional file 6: **Figure S3.** PC2 loadings for PCA analysis of microbial community dynamics (Fig. 4). Only top 30 loadings and corresponding taxa are presented here for readability. (PDF 48 kb)

## Abbreviations

BTEX: Benzene, toluene, ethylbenzene, xylen
CC: Chemical cluster
DCM: Dichloromethane
DOR: Dispersant to oil ratio
DWH: Deepwater Horizon
FID: Flame ionization detector
GC: Gas chromatography
LOD: Limit of detection
MC: Microbial cluster
MS: Mass spectometry
OTU: Operational taxonomic unit
PAH: Polycyclic aromatic hydrocarbons
PCA: Principal components analysis
TEOC: Total extractable organic carbon
WAF: Water accomodated fraction
