# Supplementary material for: Oil type and temperature dependent biodegradation dynamics - Combining chemical and microbial community data through multivariate analysis
Source: BMC Microbiol. 2018 Aug 7;18:83. doi: 10.1186/s12866-018-1221-9 (PMC6081865; doi:10.1186/s12866-018-1221-9)
Supplement: Supplementary file 1 — Table S1. Experimental design. Sampling days and number of replicates are described for all experimental analyses. The following coding system is applied: X + Y + Z, where X is number of replicates for oil dispersions in natural unfiltered seawater, Y is number of replicates for dispersions in sterilized filtered seawater, and Z is number of replicates for natural unfiltered seawater without oil. (PDF 391 kb) [file 12866_2018_1221_MOESM1_ESM.pdf]

Table S1. Experimental design. Sampling days and number of replicates are described for all experimental analyses. The following coding system is applied: X + Y + Z, where X is number of replicates for oil dispersions in natural unfiltered seawater, Y is number of replicates for dispersions in sterilized filtered seawater, and Z is number of replicates for natural unfiltered seawater without oil.

Sampling day	Particle counts (Coulter Counter)	Microbial communities (DNA extraction)	Cell counts and oxygen consumption (DAPI/MPN/O2)	Chemical analyses (GC/FID and GC-MS)	Oil:Temp
0	2 + 0 + 1	2 + 0 + 1	2 + 0 + 1	2 + 1 + 1	TROLL:13°C
7	2 + 1 + 1	2 + 0 + 1	2 + 0 + 1	2 + 1 + 1	
14	2 + 1 + 1	2 + 0 + 1	2 + 0 + 1	2 + 1 + 1	
30	2 + 1 + 1	2 + 0 + 1	2 + 0 + 1	2 + 1 + 1	
0	2 + 1 + 1	2 + 0 + 1	2 + 0 + 1	2 + 1 + 1	TROLL:5°C
6	2 + 1 + 1	2 + 0 + 1	2 + 0 + 1	2 + 1 + 1	
9	2 + 1 + 1	2 + 0 + 1	2 + 0 + 1	2 + 1 + 1	
13	2 + 1 + 1	2 + 0 + 1	2 + 0 + 1	2 + 1 + 1	
16	2 + 1 + 1	2 + 0 + 1	2 + 0 + 1	2 + 1 + 1	
30	2 + 1 + 1	2 + 0 + 1	2 + 0 + 1	2 + 1 + 1	
64	2 + 1 + 1	2 + 0 + 1	2 + 0 + 1	2 + 1 + 1	
0	2 + 1 + 1	2 + 0 + 1	2 + 0 + 1	2 + 1 + 1	GRANE:13°C
3	2 + 1 + 1	2 + 0 + 1	2 + 0 + 1	2 + 1 + 1	
7	2 + 1 + 1	2 + 0 + 1	2 + 0 + 1	2 + 1 + 1	
14	2 + 1 + 1	2 + 0 + 1	2 + 0 + 1	2 + 1 + 1	
21	2 + 1 + 1	2 + 0 + 1	2 + 0 + 1	2 + 1 + 1	
30	2 + 1 + 1	2 + 0 + 1	2 + 0 + 1	2 + 1 + 1	
64	2 + 1 + 1	2 + 0 + 1	2 + 0 + 1	2 + 1 + 1	
0	2 + 1 + 1	2 + 0 + 1	2 + 0 + 1 (only O_2)	2 + 1 + 1	GRANE:5°C
6	3 + 1 + 1	3 + 0 + 1	3 + 0 + 1	3 + 1 + 1	
9	3 + 1 + 1	3 + 0 + 1	3 + 0 + 1 (only O_2)	3 + 1 + 1	
13	3 + 1 + 1	3 + 0 + 1	3 + 0 + 1	3 + 1 + 1	
16	3 + 1 + 1	3 + 0 + 1	3 + 0 + 1 (only O_2)	3 + 1 + 1	
30	3 + 1 + 1	3 + 0 + 1	3 + 0 + 1	3 + 1 + 1	
64	3 + 1 + 1	3 + 0 + 1	3 + 0 + 1	3 + 1 + 1	
