# Supplementary material for: Oil type and temperature dependent biodegradation dynamics - Combining chemical and microbial community data through multivariate analysis
Source: BMC Microbiol. 2018 Aug 7;18:83. doi: 10.1186/s12866-018-1221-9 (PMC6081865; doi:10.1186/s12866-018-1221-9)
Supplement: Supplementary file 2 — Table S2. Calculated half-lives for targeted oil compounds. (PDF 72 kb) [file 12866_2018_1221_MOESM2_ESM.pdf]

Table S2. Calculated half-lives for targeted oil compounds

	Lag phase + half-life (days)				Lag phase (days)			
	**Troll 13°C**	**Troll 5°C**	**Grane 13°C**	**Grane 5°C**	**Troll 13°C**	**Troll 5°C**	**Grane 13°C**	**Grane 5°C**
**nC-10**	nd	nd	nd	nd	nd	nd	nd	nd
**nC-11**	nd	nd	nd	nd	nd	nd	nd	nd
**nC-12**	nd	nd	3,2	13,0	nd	nd	2,9	12,9
**nC-13**	nd	nd	3,1	9,3	nd	nd	2,9	7,9
**nC-14**	nd	9,1	3,1	9,3	nd	9,0	3,0	8,0
**nC-15**	6,6	13,1	3,1	9,1	6,3	8,3	3,0	7,7
**nC-16**	6,8	13,2	3,8	8,8	6,6	8,4	2,5	6,5
**nC-17**	6,4	14,0	3,9	8,3	5,7	8,0	2,5	5,6
**Pristane**	8,5	19,6	8,9	21,3	6,9	16,0	6,8	15,8
**nC-18**	5,6	13,3	3,8	8,2	4,0	7,9	2,6	5,6
**Phytane**	9,0	24,3	10,1	23,8	6,9	21,0	7,0	15,8
**nC-19**	6,8	13,7	3,9	8,5	6,6	8,0	2,6	5,6
**nC-20**	6,8	11,6	11,8	8,6	6,6	6,0	9,1	5,5
**nC-21**	5,1	13,4	3,9	8,4	2,8	8,2	2,7	5,5
**nC-22**	4,8	13,0	3,9	8,8	2,7	12,8	2,8	6,9
**nC-23**	5,0	13,5	4,0	8,5	2,9	8,3	2,8	5,7
**nC-24**	5,2	14,5	4,1	9,3	3,1	8,6	2,8	6,5
**nC-25**	5,6	15,1	4,4	9,8	3,6	8,6	2,8	6,5
**nC-26**	5,3	14,6	4,2	9,8	3,1	8,6	2,8	7,0
**nC-27**	5,8	15,6	4,3	10,4	4,4	8,8	2,8	5,7
**nC-28**	5,6	16,9	4,3	12,9	4,1	12,0	2,8	6,8
**nC-29**	5,8	17,9	4,5	16,4	4,1	12,3	2,8	6,8
**nC-30**	6,3	17,9	4,7	16,9	4,8	15,0	2,7	7,1
**nC-31**	6,9	16,3	4,9	20,2	6,6	15,9	3,0	10,2
**nC-32**	6,9	16,4	6,8	20,1	6,7	15,9	6,6	11,6
**nC-33**	6,9	16,7	5,1	20,2	6,7	16,0	2,7	12,2
**nC-34**	0,1	16,7	6,8	16,5	0,0	16,0	6,5	15,9
**nC-35**	0,1	16,3	6,8	19,1	0,0	15,9	6,5	12,6
**nC-36**	0,1	16,5	6,7	18,8	0,0	15,9	6,5	12,1

	Lag phase + half-life (days)				Lag phase (days)			
	**Troll 13°C**	**Troll 5°C**	**Grane 13°C**	**Grane 5°C**	**Troll 13°C**	**Troll 5°C**	**Grane 13°C**	**Grane 5°C**
**SUM n-alkanes**	6,669	16,9	4,5	10,3	5,0	11,1	2,6	5,4
**Naphthalene**	1,0	13,4	3,6	13,0	0,0	8,9	3,0	8,4
**C4-naphthalenes**	5,8	16,2	7,3	16,0	5,0	15,8	5,4	8,5
**Fluorene**	0,1	15,9	6,5	15,7	0,0	14,6	6,3	14,2
**C1-fluorenes**	0,1	16,2	6,9	15,1	0,0	15,8	6,7	7,4
**C2-fluorenes**	0,1	16,4	7,1	18,0	0,0	15,9	6,9	10,6
**C3-fluorenes**	7,2	16,5	14,0	23,1	7,0	16,0	13,8	10,2
**Phenanthrene**	0,1	13,1	3,6	14,1	0,0	13,0	3,0	12,9
**C1-phenanthrenes/anthracenes**	0,1	14,9	6,9	15,6	0,0	13,8	6,7	8,4
**C2-phenanthrenes/anthracenes**	7,0	16,4	8,2	18,4	6,8	15,9	6,4	10,9
**C3-phenanthrenes/anthracenes**	14,1	30,3	14,8	35,3	13,5	29,2	11,5	10,8
**C4-phenanthrenes/anthracenes**	15,0	40,1	17,5	54,6	13,8	25,3	13,0	11,3
**Dibenzothiophene**	0,3	16,2	6,4	16,6	0,0	15,8	6,2	15,3
**C1-dibenzothiophenes**	21,3	16,3	7,0	24,1	6,0	15,8	6,7	8,5
**C2-dibenzothiophenes**	6,9	17,9	7,5	18,8	6,7	16,0	6,9	11,1
**C3-dibenzothiophenes**	14,3	30,3	15,2	37,7	6,1	29,2	11,5	8,9
**C4-dibenzothiophenes**	14,5	40,5	18,1	68,2	14,0	26,5	13,3	0,0
**Fluoranthene**	7,2	28,8	7,2	28,2	7,0	27,6	7,0	24,5
**Pyrene**	7,0	18,8	7,2	21,6	6,8	16,0	7,0	16,0
**C1-fluoranthrenes/pyrenes**	11,9	30,0	15,8	32,0	4,4	28,9	9,4	6,5
**C2-fluoranthenes/pyrenes**	22,3	54,0	nd	77,5	6,9	10,7	nd	0,0
**C3-fluoranthenes/pyrenes**	24,8	56,8	nd	119,4	16,9	25,4	nd	0,0
**Chrysene**	15,3	30,8	14,1	30,9	13,9	29,7	13,9	29,9
**C1-chrysenes**	14,4	30,9	18,8	88,4	14,0	29,9	12,4	3,2
**C2-chrysenes**	28,0	34,4	27,3	127,7	19,1	30,0	17,2	0,0
**C3-chrysenes**	37,0	100,8	nd	650,9	15,6	39,6	nd	0,0
**SUM PAH**	5,7	16,3	9,3	18,4	2,4	12,9	5,8	10,1
**n-C5 (Pentane)**	0,1	19,5	5,9	15,9	0,0	16,0	5,2	14,4
**n-C6 (Hexane)**	0,1	14,3	3,0	6,1	0,0	9,9	2,9	5,8
**n-C7 (Heptane)**	0,2	1,1	0,0	5,9	0,0	0,0	0,0	5,8

	Lag phase + half-life (days)				Lag phase (days)			
	**Troll 13°C**	**Troll 5°C**	**Grane 13°C**	**Grane 5°C**	**Troll 13°C**	**Troll 5°C**	**Grane 13°C**	**Grane 5°C**
**n-C8 (Octane)**	nd	nd	0,1	nd	nd	nd	0,0	nd
**n-C9 (Nonane)**	nd	nd	0,1	0,0	nd	nd	0,0	0,0
**n-C10 (Decane)**	0,3	nd	6,9	19,6	0,0	nd	6,7	13,0
**Benzene**	7,2	16,7	7,0	17,8	7,0	16,0	6,8	16,0
**Toluene**	6,7	16,4	6,2	16,3	4,5	15,9	5,0	15,9
**Ethylbenzene**	0,1	16,2	5,0	16,1	0,0	15,8	4,3	15,6
**m-Xylene**	0,1	13,7	3,8	14,2	0,0	13,0	3,0	12,8
**p-Xylene**	0,1	14,1	3,7	14,6	0,0	13,1	3,0	13,5
**o-Xylene**	0,1	16,2	6,4	16,1	0,0	15,8	6,2	15,7
**Propylbenzene**	0,1	16,1	3,7	14,9	0,0	14,8	3,0	12,4
**n-Butylbenzene**	0,1	14,2	3,2	13,9	0,0	12,7	3,0	12,1
**n-Pentylbenzene**	nd	nd	nd	nd	nd	nd	nd	nd
**SUM VOC**	7,0	16,2	3,0	16,1	6,8	15,8	2,9	15,7
