# Supplementary material for: Oil type and temperature dependent biodegradation dynamics - Combining chemical and microbial community data through multivariate analysis
Source: BMC Microbiol. 2018 Aug 7;18:83. doi: 10.1186/s12866-018-1221-9 (PMC6081865; doi:10.1186/s12866-018-1221-9)
Supplement: Supplementary file 3 — Table S3. List of abbreviations for aromatics compounds used in correlation plots. (PDF 40 kb) [file 12866_2018_1221_MOESM3_ESM.pdf]

Table S3. List of abbreviations for aromatics compounds used in correlation plots

Group	Compound	Abb
Naphthalenes	Naphthalene	N
	C1-naphthalenes	N1
	C2-naphthalenes	N2
	C3-naphthalenes	N3
	C4-naphthalenes	N4
2-3 ring PAHs	Biphenyl	B
	Acenaphthylene	ANY
	Acenaphthene	ANA
	Dibenzofuran	DBF
	Fluorene	F
	C1-fluorenes	F1
	C2-fluorenes	F2
	C3-fluorenes	F3
	Phenanthrene	P
	C1-phenanthrenes/anthracenes	P1
	C2-phenanthrenes/anthracenes	P2
	C3-phenanthrenes/anthracenes	P3
	C4-phenanthrenes/anthracenes	P4
	Dibenzothiophene	D
	C1-dibenzothiophenes	D1
	C2-dibenzothiophenes	D2
	C3-dibenzothiophenes	D3
	C4-dibenzothiophenes	D4
4-6 ring PAHs	Fluoranthene	FL
	Pyrene	PY
	C1-fluoranthrenes/pyrenes	FL1
	C2-fluoranthenes/pyrenes	FL2
	C3-fluoranthenes/pyrenes	FL3
	Benz[*a*]anthracene	BA
	Chrysene	C
	C1-chrysenes	C1
	C2-chrysenes	C2
	C3-chrysenes	C3
	C4-chrysenes	C4
