# Supplementary material for: Oil type and temperature dependent biodegradation dynamics - Combining chemical and microbial community data through multivariate analysis
Source: BMC Microbiol. 2018 Aug 7;18:83. doi: 10.1186/s12866-018-1221-9 (PMC6081865; doi:10.1186/s12866-018-1221-9)
Supplement: Supplementary file 4 — Figure S1. Microbial community composition in control samples over the incubation period. (PDF 103 kb) [file 12866_2018_1221_MOESM4_ESM.pdf]

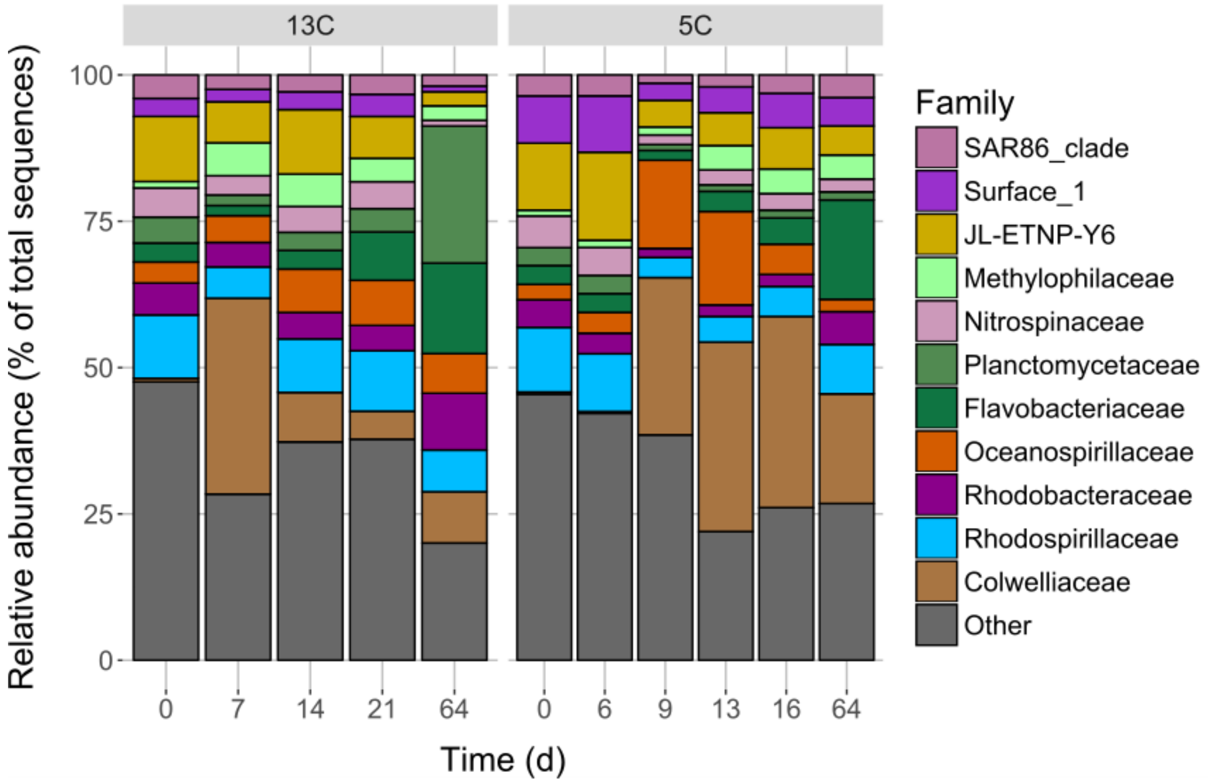

Figure S1. Microbial community composition in control samples for both temperatures (5 °C and 13 °C) over the incubation period of 64 days.
