# Supplementary material for: Oil type and temperature dependent biodegradation dynamics - Combining chemical and microbial community data through multivariate analysis
Source: BMC Microbiol. 2018 Aug 7;18:83. doi: 10.1186/s12866-018-1221-9 (PMC6081865; doi:10.1186/s12866-018-1221-9)
Supplement: Supplementary file 5 — Figure S2. Biotransformation of targeted semivolatile n-alkanes (A and B) and PAH (C and D) in dispersions of Troll and Grane oils at 13 °C and 5 °C. (PDF 195 kb) [file 12866_2018_1221_MOESM5_ESM.pdf]

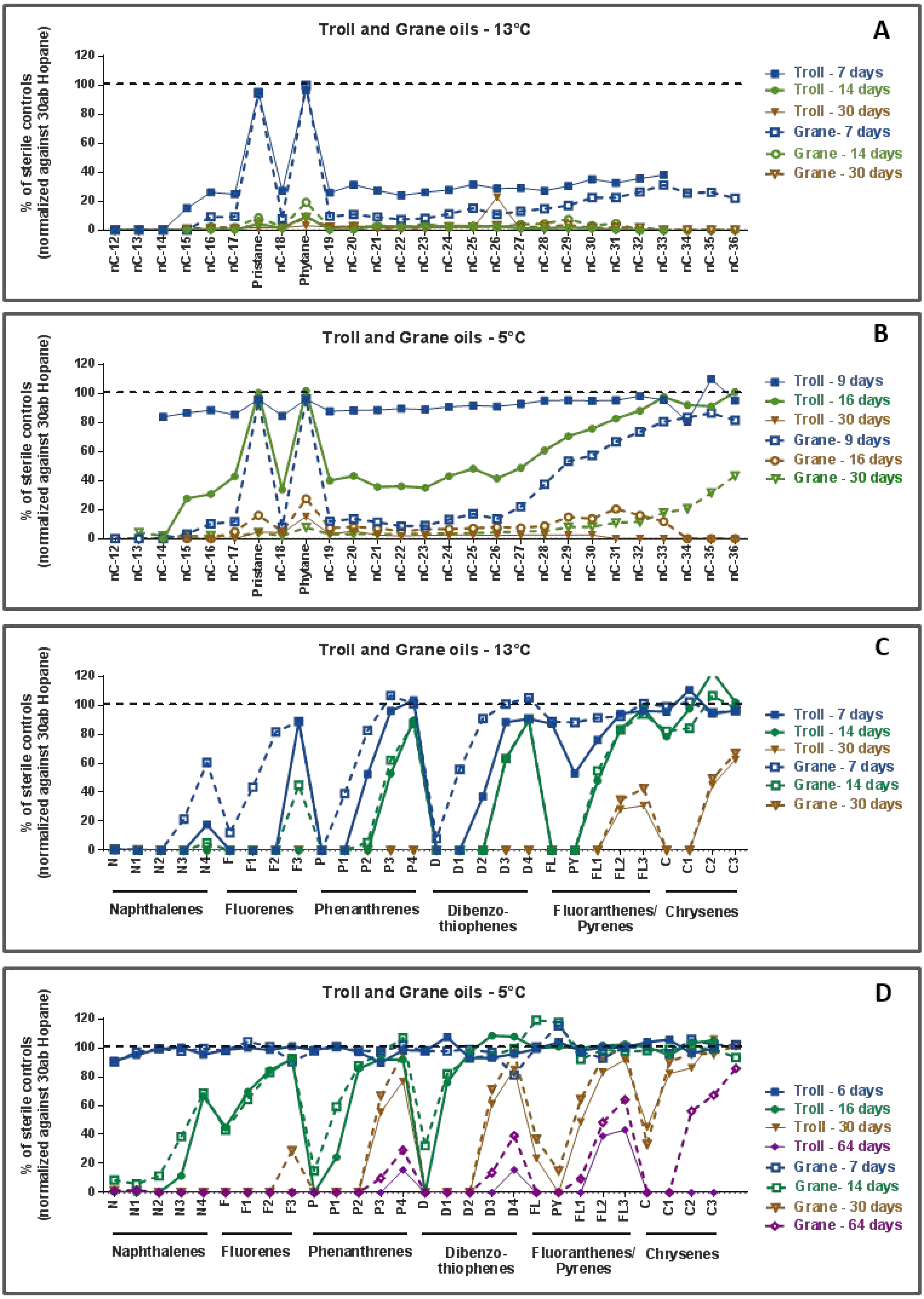

Figure S1. Biotransformation of targeted semivolatile n-alkanes (A and B) and PAH (C and D) in dispersions of Troll and Grane oils at 13 °C and 5 °C.
