# Supplementary material for: Oil type and temperature dependent biodegradation dynamics - Combining chemical and microbial community data through multivariate analysis
Source: BMC Microbiol. 2018 Aug 7;18:83. doi: 10.1186/s12866-018-1221-9 (PMC6081865; doi:10.1186/s12866-018-1221-9)
Supplement: Supplementary file 6 — Figure S3. PC2 loadings for PCA analysis of microbial community dynamics (Fig. 4). Only top 30 loadings and corresponding taxa are presented here for readability. (PDF 48 kb) [file 12866_2018_1221_MOESM6_ESM.pdf]

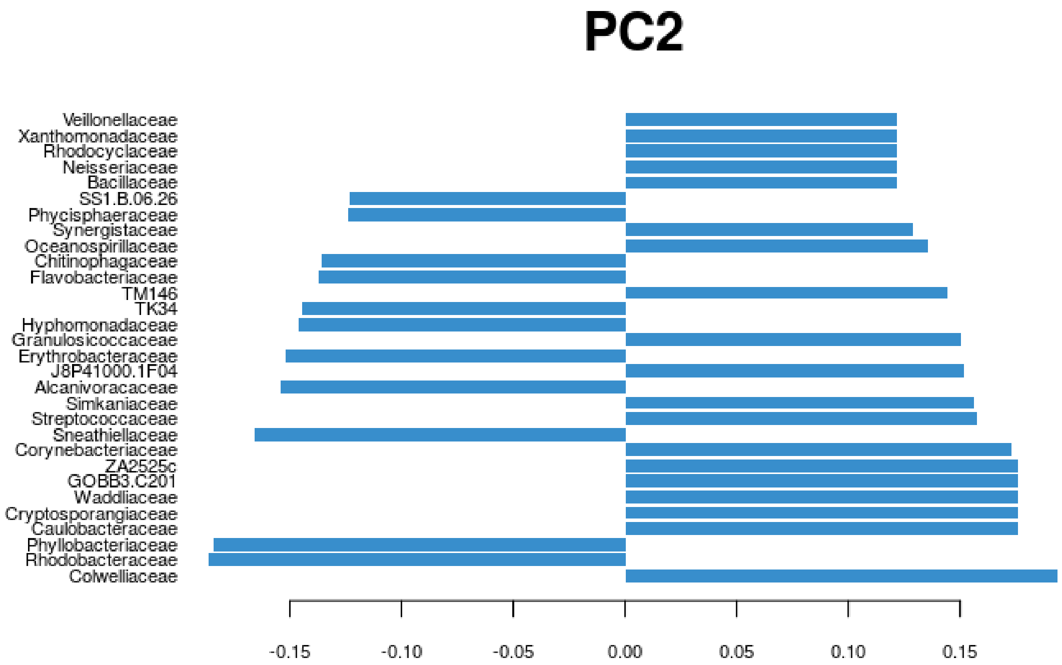

Figure S1. PC2 loadings for PCA analysis of microbial community dynamics (Figure 4). Only top 30 loadings and corresponding taxa are presented here for readability.
